# Characteristics and expectations among emergency department patients in India

**DOI:** 10.1371/journal.pgph.0000009

**Published:** 2022-02-11

**Authors:** Kevin Davey, Sumin Jacob, Nilesh Prasad, Manjula Shri, Richard Amdur, Janice Blanchard, Jeffrey Smith, Katherine Douglass

**Affiliations:** 1 Department of Emergency Medicine, George Washington University, Washington, DC, United States of America; 2 Department of Emergency Medicine, Believer’s Church Medical College Hospital, Thiruvalla, Kerala, India; 3 Department of Emergency Medicine, Max Super Specialty Hospital Patparganj, Patparganj, Delhi, India; 4 Meenakshi Mission Hospital and Research Center, Institute of Emergency Medicine, Madurai, Tamil Nadu, India; University of Milano–Bicocca: Universita degli Studi di Milano-Bicocca, ITALY

## Abstract

In India, and many low-middle income countries (LMICs), emergency medicine (EM) remains a poorly defined specialty and an unregulated field of clinical practice. Recognition of the attitudes, understanding, and expectations of patients presenting to Indian EDs will be crucial to the continued development of EM as a specialty. This is a multicenter, prospective, cross sectional study of adult and pediatric patients presenting to the ED in three geographically distinct regions of India. Participants were surveyed about their expectations regarding the type of care that they expected to receive, previous treatment options they have utilized, basic understanding of ED operations, factors contributing to their decision to seek ED care, and basic demographic information. 779 patients were approached to participate in the study, of which 698 (90%) completed the survey. Common ways that patients reported learning about the ED were referral from another healthcare provider (45%) and recommendation by a family member (61%). Participants chose the ED was because they thought they would be seen quickly (89%), would receive acute pain management (45%), their regular outpatient care was closed (45%), or were sent by another doctor (45%). Patients expected to wait 0.3 hours (18 minutes) on average to see a doctor in the ED. Over 75% or patients expected to see a specialist consultant in the ED and 12% expected to see their personal physician. Eighty-five percent of patients were triaged as moderate or high acuity, and 74% of patients were admitted. This study found that ED in India is utilized by a population with an extremely high acuity of medical illness that attempts to access healthcare through multiple avenues. Patients most frequently visit the ED due to a referral from another healthcare provider or family member. Most patients are aware of the existence of the ED, though understanding of available services may be lacking. Future research should focus on community outreach and education initiatives on ED services.

## Introduction

In India, and many low-middle income countries (LMICs), emergency medicine (EM) remains a poorly defined specialty and an unregulated field of clinical practice [1,2]. In these settings the notion of the emergency department (ED) as a place for the treatment of the acutely ill or injured remains largely a new concept. A better understanding of patient characteristics as well as recognition of the attitudes, understanding, and expectations of the ED population in India will be crucial to the continued development of EM as a specialty. Prior studies conducted in LMIC’s have found both logistical and cultural barriers to accessing emergency care [3–7]. While these studies give some insight into the obstacles to accessing emergency care in LMICs, small sample sizes, single centers studies, and the overall the dearth of data, often limit generalizability. Furthermore, while prior studies have evaluated barriers to care, few have thus far sought to evaluate the expectations and understanding of available care in the ED population.

The majority of studies conducted around ED utilization in more developed countries have focused on frequent utilizers and those who present to the ED for non-emergent conditions. While it is often believed that a lack of access to primary care drives recurrent ED utilization in more developed settings, the data surrounding this theory is mixed [8–13]. Data does demonstrate that patients seeking emergency care do so for a variety of reasons including the perceived severity of their illness, and the belief that they will be seen and get test results quickly, as well as transportation barriers and long wait times at their primary care offices [14–16]. While this may give some insight into the behaviors of the ED population in certain contexts, the differences in healthcare systems and population demographics between countries make direct comparisons of their ED populations difficult.

Our institution has been working in India since 2006, in partnership with local hospitals to build education and training programs in EM [17]. During that time, we have witnessed significant variability among patients at different institutions in their expectations and responses to their experience and care in the ED. The objective of this study was to better understand the characteristics and expectations of patients presenting to the EDs in India. We are aware of no prior studies which have sought to evaluate these aspects of the Indian ED population. These findings will provide the context of local norms and traditions to which future efforts to maximize appropriate utilization, improve patient education, and expand access to emergency care can be tailored.

## Materials and methods

This was a multicenter, prospective, cross sectional study of adult and pediatric patients and their families presenting to Indian EDs between April 1, 2019 and March 31, 2020 at three private hospitals in geographically distinct regions of India. Hospital details can be found in Table 1.

**Table 1:** Hospital details.

Hospital	Location	Number of Patients Enrolled (n = 779)	Average Annual ED Volume (patients)
Meenakshi Mission Hospital and Research Center	Madurai, Tamil Nadu	303 (38.9%)	25,000
Max Super Specialty Hospital Patparganj	Patparganj, Delhi	220 (28.2%)	30,000
Believer’s Church Medical College Hospital	Thiruvalla, Kerala	256 (32.9%)	25,000

Upon arrival to the ED, patients were recruited for voluntary participation using a random number generator. Patients who were assigned an even number were approached to participate in the study. Once a patient was identified for potential enrollment a member of the study team presented them with an informed consent document explaining the study, as well as the potential risks and benefits of participation (S1 File). The informed consent was read aloud by the study team member. Patients were also provided their own copy to review. After reading the document patients were asked by the study team member if they wished to participate in the study. Those who answered “yes” were given a survey to complete. The survey was administered by ED staff who received training on survey technique prior to the beginning of the study. ED staff members enrolled patients only when they were not working clinically and not involved in the patient’s care. Participants were given a survey to complete at the beginning of the ED visit. The survey queried patients about their expectations regarding the type and quality of care that they expected to receive in the ED, what previous treatment options they have utilized prior to coming to the ED, their basic understanding of ED flow and operations, source of knowledge about the ED, factors contributing to their decision to seek ED care, as well as basic demographic information. No personal identifying information was collected.

**Fig 1:**
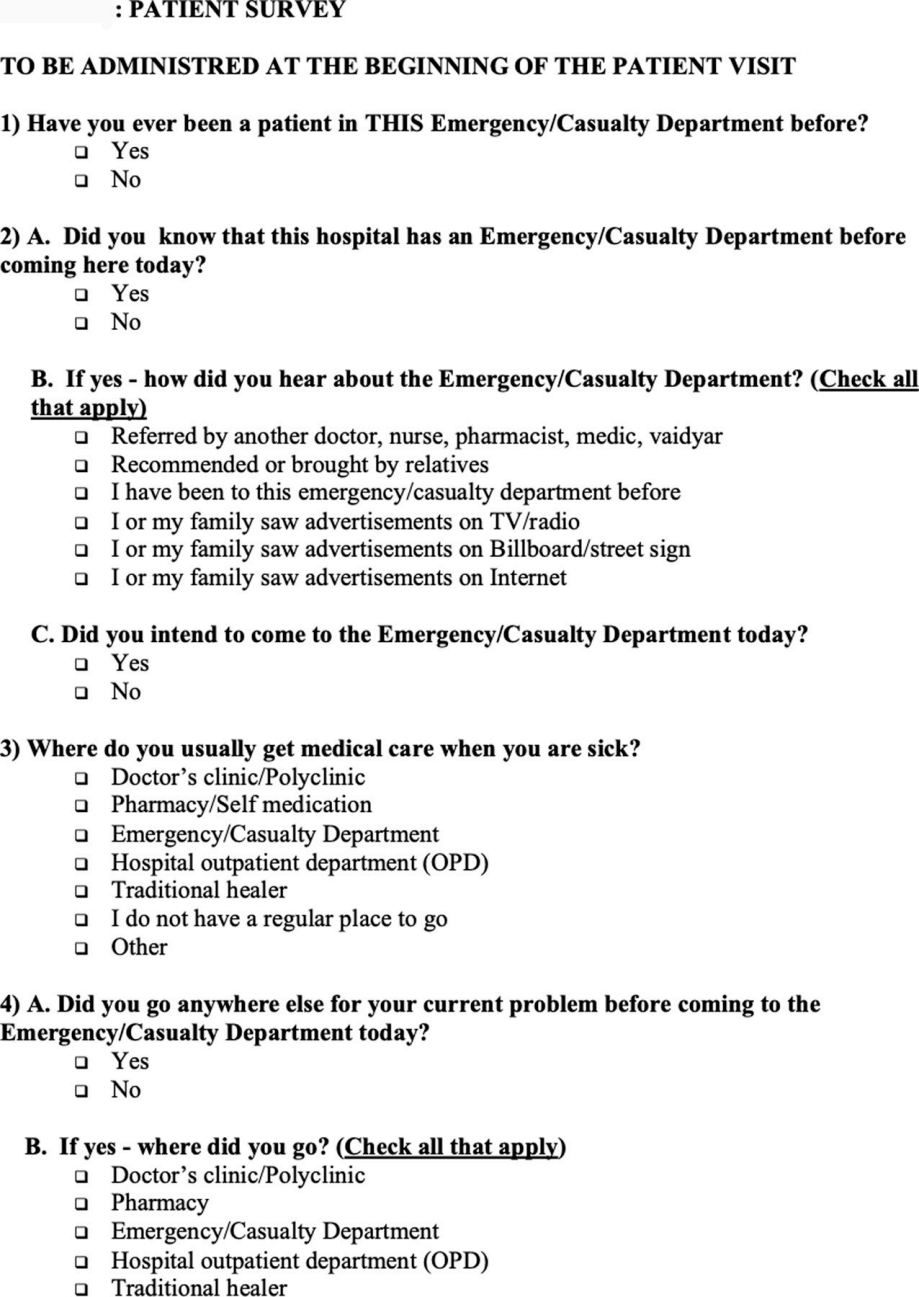
Survey tool.

The survey was developed for this study and piloted prior to beginning the study. The survey was available in English, Hindi, Kannada, Malayalam, Tamil, and Tegulu. A copy of the survey can be found in Fig 1. For pediatric patients, consent was taken from an accompanying adult (≥18 years old) parent or guardian, who was asked to complete the survey on the patients’ behalf. Exclusion criteria included acute intoxication, altered mental status, severe medical illness that prohibited completion of the survey, as well as any incarcerated status. Some patients who met exclusion criteria with altered mental status or severe medical illness, may have been enrolled in the study if they presented to the ED with accompanying family members, who completed the survey on their behalf.

Data was collected and entered contemporaneously into a REDCap database online storage system hosted at George Washington University [18,19]. REDCap (Research Electronic Data Capture) is a secure, web-based software platform designed to support data capture for research studies. Data analysis consisted of basic descriptive statistics and comparisons of matched-question answers using raw numbers, reporting of raw differences, and differences in raw proportions in the case of binary outcomes. Chi-square analysis was used to compare differences in proportions between groups using 95% confidence intervals.

### Ethics statement

The study was approved by the George Washington University Institutional Review Board (IRB number: 180680) as well as the ethics committees of Meenakshi Mission Hospital and Research Centre, Believer’s Church Medical College Hospital, and Max Superspecialty Hospital Patparganj (S3 File). Verbal consent was obtained from all participants prior to enrollment in the study.

## Results

779 patients were approached to participate in the study, of which 698 (90%) completed the survey. Details on patient demographics and characteristics can be found in Table 2. Eighty-five percent of patients were triaged as moderate or high acuity, and 74% of patients were admitted.

**Table 2:** Patient demographics.

	Patient Sex/Gender
Male	470 (60.3%)
Female	309 (39.7%)
	Patient Age (years)
Mean	47.7
Median	51
Range	0 to 95
	Patient Triage Category
Red	425 (54.6%)
Yellow	236 (30.3%)
Green	118 (15.1%)
	Patient Mode of Transportation
Ambulance	268 (34.4%)
Private Vehicle	428 (54.9%)
Public Bus	33 (4.2%)
On Foot	50 (6.4%)
	Patient Disposition
Admitted ICU	285 (36.6%)
Admitted Ward	197 (25.3%)
Voluntary Transfer	48 (6.2%)
Transfer AMA	47 (6.0%)
Left (home) AMA	44 (5.6%)
Discharged	141 (18.1%)
Died	17 (2.2%)
	Patient Level of Education
Less than high school	126 (18.1%)
High school graduate	109 (21.4%)
Some college	149 (21.4%)
College Graduate	190 (27.3%)
Post Graduate	118 (16.9%)
Don’t Know	5 (0.7%)

Two hundred fifty seven (37%) of patients reported having previously been a patient in the ED to which they presented. About half of patients reported that their visit to the ED was unplanned. Over 90% of patients were aware that their hospital had an ED. The most common that patients reported learning about the ED were referral from another healthcare provider (45%) and recommendation by a family member (61%). (Table 3). Three hundred forty three patients (49%) reported seeking care elsewhere for their current problem prior to coming to the ED (Table 4). Commonly reported locations where patients previously sought care included an outpatient doctors’ clinic (35%), a hospital-based outpatient department (19%), and a different ED (61%). The most common reason participants chose the ED was because they thought they would be seen quickly (89%), for acute pain management (45%), because their regular outpatient care was closed (45%), because they were sent by their doctor (45%) (Table 5).

**Table 3:** How patients learned about the emergency department.

How did you learn about the ED?	Number of Responses (n = 698)
Recommended by relatives	442 (60.5%)
Referred by another healthcare provider (doctor, nurse, pharmacist, medic, vaidyar)	316 (45.3%)
I have been a patient in this Emergency/Casualty Department before	252 (36.1%)
I or my family saw an advertisement on a billboard/street sign	173 (24.8%)
I or my family saw an advertisement on the internet	127 (18.2%)
I or my family saw an advertisement on TV/Radio	114 (16.3%)

**Table 4:** Where did you seek care for your current problem prior to coming to the ED?.

Location	Reponses (n = 343)
A different Emergency/Casualty Department	209 (60.9%)
Doctors clinic/polyclinic	119 (34.7%)
Hospital outpatient clinic	66 (19.2%)
Pharmacy/self-medication	25 (7.3%)
Other	24 (7.0%)
Traditional Healer	9 (2.6%)

**Table 5:** Reasons for choosing the ED.

Reasons for choosing the ED	Reponses (n = 698)
Belief they would be seen quickly	617 (88.5%)
Pain Treatment	316 (45.3%)
Regular outpatient clinic closed	311 (44.6%)
Referred by another doctor	311 (44.6)
No other option for care	227 (32.6%)
High Quality Care	199 (28.6%)

Patients reported getting their regular medical care from a number of different locations, most commonly a hospital-based outpatient clinic (40%) or a non-hospital based outpatient polyclinic (44%). Only 6% of patients reported getting their regular care at the ED (Table 6). One hundred sixty-six patients (24%) reported having been a patient in the hospital of the ED they were visiting in the past week. One third of these (33%) had been admitted to the ward. Another 30% had been patients in the ED (Table 7). Patients in this study reported expecting to wait 0.3 hours (18 minutes) on average to see a doctor in the ER, 1.5 hours (90 minutes) for laboratory results, and less than 3 hours for all imaging results (Table 8). Over 75% or patients in our study expected to see a specialist consultant in the ED and 12% expected to see their personal physician (Table 9).

**Table 6:** Where do you usually get your medical care?.

Where do you usually seek care?	Reponses (n = 698)
Doctors clinic/polyclinic	307 (44%)
Hospital outpatient clinic	277 (39.7%)
Emergency/Casualty Department	44 (6.3%)
Pharmacy/self-medication	36 (5.2%)
I do not have a regular place to go for care	19 (2.7%)
Traditional Healer	14 (2%)
Other	1 (0.1%)

**Table 7:** If you were a patient in this hospital in the past week, where were you seen?.

Hospital Location	Reponses (n = 166)
Other	88 (53%)
Admitted to Ward	55 (33.1%)
Emergency/Casualty Department	50 (30.1%)
Medicine Outpatient Clinic	28 (16.9%)
Surgery Outpatient Clinic	12 (7.2%)
Pediatrics Outpatient Clinic	12 (7.2%)
OB-GYN Outpatient Clinic	5 (3%)
Psychiatry Outpatient Clinic	1 (0.6%)

**Table 8:** Expected wait times.

Expected wait times (hrs)	Mean	Range
To see an ER doctor	0.31	0.16–5.0
To see a specialist doctor	1.30	0.83–12.0
For your laboratory/blood reports	1.42	0.25–22.9
For your imaging results	2.85	0.83–24
To go to the ward	2.22	0.25–12

**Table 9:** Expected provider in the ED.

Expected Provider	Responses (n = 698)
Nurse	246
ER/Casualty doctor	584
Specialty consultant	525
My personal doctor	83
Other	8

A subgroup analysis was also conducted of patients who arrived by ambulance vs those who used other modes of transportation, as these patients may represent a unique cohort who presented as the result of an unexpected accident or medical emergency and decisions regarding where to seek care may have been different than those who used another mode of transportation. There was no statistically significant difference between these groups when asked whether they intended to come to the emergency room (45.9% vs 52.3%, p = 0.14). Patients who presented by ambulance were more like to say they had sought care from another source prior to coming to the ED (82.1% vs 33.8%, (p<0.05), and had been referred to the ED by another doctor (p<0.05) (Table 10).

**Table 10:** Ambulance vs other mode of transport.

	Ambulance (n = 224)	Other mode of Transportation (n = 474)	Difference, (95% CI, P-value)
Intended to come to the ED?	45.9%	52.3%	6.4% (1.9–14.6, p = 0.14)
Sought care elsewhere prior to the ED?	82.1%	33.8%	48.3% (41.2–54.4, p < 0.05)
**Reason for choosing ED**			
High Quality Care	41.5%	22.6%	18.9% (11.4–26.3, p<0.05)
Pain Treatment	33.9%	50.8%	16.9% (9.1–24.3, p<0.05)
Belief they would be seen quickly	88.8%	88.4%	0.4% (-5.1–5.1, p = 0.88)
Regular outpatient clinic closed	37.9%	44.7%	6.8 (-1.1–14.4, p = 0.09)
Referred by another doctor	62.1%	36.5%	24.7%, (16.8–32.1, p<0.05)
No other option for care	29.5%	34.2%	4.7% (-2.8–11.8, p = 0.22)
Other	8.5%	2.7%	5.8% (2.3–10.3, p<0.05)

## Discussion

As the field of EM continues to develop in India and other LMICs, understanding the characteristics and expectations of the ED population will be crucial to guiding patient education, targeting community outreach, and improving access to care. This study represents the first such multicenter analysis amongst ED patients in India.

Data on ED utilization from more developed settings demonstrate that the ED population have more chronic medical problems, are of lower socioeconomic status, seek care from multiple venues, and do so at a higher rate than the general population [13,15,20–22]. There are similarities between these findings and those of the current study. Nearly a quarter of patients from the current study reported being a patient in the hospital to which they presented in the past week, two-thirds of which had been admitted or seen in the ED, suggesting the presence of some form of pre-existing medical condition, or an ongoing condition that had continued to worsen. Patients also reported having sought care from other sources, and most patients in our sample reported previously seeking care in an ED for the current medical problem. Eighty five percent of patients were triaged as high or moderate acuity, and 74% were admitted to the hospital, over half of which went to the ICU. Taken together, this suggests that the ED in India is utilized by a consistent patient population with comorbid medical conditions that usually require a higher level of care when they end up using the ED. Whether these characteristics are indicative of a patient population with poorly controlled chronic medical problems at baseline, overall high utilization of medical resources, or some combination of factors remains to be determined, and should be the focus of future studies.

Patients learned about the ED through a variety of avenues. The most frequently cited reason for choosing the ED was the referral or recommendation by a family member or healthcare provider. Patients reported multiple reasons for choosing the ED including pain control, the belief that they would be seen by a doctor quickly, referral by another healthcare provider, and a lack of other healthcare options. These findings are consistent with those from more developed settings [13,14]; however, one key difference is that almost half of patients in the current study reported coming to the ED because their primary care clinic was closed, and one-third reported having no other option for seeking care. These numbers are substantially higher than in more developed settings [12,14,23]. The reasons for these findings are likely multifactorial. While for many people in India access to medical care is limited, that is less true for those who reside in urban centers and utilize the private healthcare system, where this study was conducted [24,25]. Furthermore, the outpatient clinic system in many Indian hospitals, both public and private, is robust, and acts as the primary access point for patients into the healthcare system [24,25]. This may explain why most patients in this study indicated that they usually get their healthcare from some form or primary care or outpatient clinic, and why commonly cited reasons for choosing the ED were that their regular outpatient clinic was closed or that they were referred by another doctor. It may also explain the high level of acuity among ED patients in this study, as many of the low acuity cases that might be seen in the ED in places like the United States, are instead treated by outpatient providers. Future studies including public sector hospitals, as well as national metrics that track ED utilization may help clarify these findings.

A subgroup analysis was conducted of patients who arrived by ambulance vs those who used other modes of transportation. These patients may represent a unique cohort who presented as the result of an unexpected accident or medical emergency and one might expect that decisions and rationale around care seeking may have been different in this group. Interestingly, when asked whether they intended to come the ED we found no statistically significant difference between these groups. In fact, among the significant differences we did find, were that patients who arrived by ambulance were more likely to have sought care somewhere else for their current medical problem prior to coming to the ED (82.1% vs. 33.8%, p<0.05) and were more likely to say they chose the ED because they were sent by another doctor (62.1% vs 36.5%, p<0.05). The reason for these findings are likely related to the structure of EMS services in India. There is no centralized EMS system in India, and few public ambulances available to serve India’s large population. As a result, private ambulance services, frequently affiliated with local hospitals, have moved to fill the gap. Unfortunately, these ambulances usually have little or no coordination with local police or fire departments [26]. In private hospitals like the ones where this study was conducted, ambulances are largely used to transfer patients between hospitals, rather than responding to emergencies within the community. It is likely that the cohort of patients who came by ambulance in the current study represent patients who were either transferred from another facility for a higher level of care, or called the ambulance with the intention to come to the specific hospital to which they presented, rather than patients experiencing unexpected emergencies in the community.

There is no centralized organization in India that tracks ED visits. National data on ED wait times, length of stay, frequency of specialist consultation and other metrics is limited, making it difficult to compare the reported expectations from this study with reality. Studies from other LMICs have shown findings similar to those described here, with patients expecting to wait less than 30 minutes to see an ED provider, and less than 20 minutes to see a specialty consultant [27,28]. Studies on expectation in more developed countries, also find that patients generally expect to be seen promptly and for results to be made available quickly [29,30]. The CDC reports that the average wait time to see a provider the U.S. is 37.5 minutes and data from other studies on ED wait times report a total length of stay between 2 and 4 hours [20,31,32]. While there is no data from India for comparison, US data show that specialty consultation occurs for less than 10% of ED visits [20]. Three quarters of patients (75%) in the current study reported expecting to see a specialist consultant during their ED visit, and 12% reported expecting to see their personal physician in the ED. While these findings may sound alarming, considering the newness of EM in India, and the high rate of physician referral to the ED among patients in this study (45%), significant systems differences may be impacting this data.

Interpretation of findings around patient expectations is difficult due to a lack of standardized metrics around how to measure expectations, as well as differing cultural context between the healthcare and emergency care settings of different countries. In order to put findings around patient expectations of ED care in the proper context, more data on ED metrics would be helpful to see how patients’ expectations of care compare with reality. The lack of existing data is most likely a reflection of the underdevelopment of the specialty in India and the subsequent lack of centralized and regional agencies that track ED visits. Further differentiation between the public and private sector may also be relevant. In the meantime, continued patient education and outreach efforts regarding the available services in the ED will be necessary.

### Limitations

The current study was conducted in private, urban hospitals that may charge higher fees and see a greater proportion of patients with private insurance than public sector hospitals. As such, there may be a selection bias towards patients of higher socioeconomic status. The study may not be generalizable to the ED population seen in public hospitals, or more rural settings.

The EDs in this study use a 3-tiered triage system rather than the 5 tier Emergency Severity Index (ESI) system use in many more developed healthcare systems. While this somewhat complicates attempts at comparisons of acuity between systems, it is unlikely that this discrepancy alone accounts for the substantial difference in acuity and admissions rates between higher resource settings and those found in this study.

This study did not directly assess the medical reason for presentation to the ED, nor the existence of comorbid health problems among ED patients. As such, we are unable to make definitive statements about the pre-existing health problems of the ED population.

## Conclusion

The current study demonstrates that the ED in India is utilized by a population with an extremely high acuity of medical illness that attempts to access healthcare through multiple avenues, and frequently requires a higher level of care at the time of their ED visit. These findings exemplify the need for increased access to emergency care services in India. Patients most frequently visited the ED due to a referral from another trusted healthcare provider or family member, and in the absence of other options for attaining care. Most patients are aware of the existence of the ED, though understanding of available services may be lacking. Future research should focus on complaint specific reasons for ED presentation, the prevalence of chronic medical problems in the Indian ED population, community outreach and education initiatives on ED services, and should include public hospitals. More detailed studies exploring the factors driving ED utilization in India and other LMICs will be of value to policy makers and may guide improved distribution of healthcare resources.

## Supporting information

S1 File Informed consent. (DOCX)

S2 File Questionnaire on inclusivity in global research. (DOCX)

S3 File Ethics committee approval. (PDF)

S1 Data (XLSX)

## References

[1] Jamison D; Breman J; Measham A; Alleyne G; Claeson M; Evans D, et al. World Bank. Disease Control Priorities Project. Disease Control Priorities in Developing Countries. New York, NY: Oxford University Press, 2006. 21250309

[2] Sriram V; Hyder A; Bennett S. “The Making of a New Medical Specialty: A Policy Analysis of the Development of Emergency Medicine in India.” *International Journal of Health Policy and Management*, vol. 7, no. 11, 2018, pp. 993–1006., doi: 10.15171/ijhpm.2018.55 30624873 PMC6326640

[3] Wilson A; Hillman S; Rosato M; Skelton J; Costello A; Hussein J; et al. A systematic review and thematic synthesis of qualitative studies on maternal emergency transport in low-and middle-income countries. Int J Gynecol Obstet. 2013;122(3):192–201. doi: 10.1016/j.ijgo.2013.03.030 23806250

[4] Kobusingye O; Hyder A; Bishai D; Hicks E; Mock C; Joshipura M. Emergency medical systems in low- and middle-income countries: recommendations for action. Bull World Health Organ. 2005;83:626–631. doi: /S0042-96862005000800017 16184282 PMC2626309

[5] Jammeh A; Sundby J; Vangen S. Barriers to emergency obstetric care services in perinatal deaths in rural Gambia: a qualitative in-depth interview study. ISRN Obstet Gynecol. 2011;2011:981096. doi: 10.5402/2011/981096 21766039 PMC3135215

[6] Cham M; Sundby J; Vangen S. Maternal mortality in the rural Gambia, a qualitative study on access to emergency obstetric care. Reprod Health. 2005;2:3. doi: 10.1186/1742-4755-2-3 15871743 PMC1142340

[7] Essendi H; Mills S; Fotso J. Barriers to formal emergency obstetric care. services’ utilization. J Urban Health. 2011;88(Suppl 2):S356–69. doi: 10.1007/s11524-010-9481-1 20700769 PMC3132235

[8] Flores-Mateo G; Violan-Fors C; Carrillo-Santisteve P; Peiró S; Argimon J. Effectiveness of organizational interventions to reduce emergency department utilization: a systematic review. PloS one. 2012;7(5):e35903 doi: 10.1371/journal.pone.0035903 22567118 PMC3342316

[9] Morgan S; Chang A; Alqatari M; Pines J. Non–Emergency Department Interventions to Reduce ED Utilization: A Systematic Review. Acad Emerg Med. 2013;20(10):969–85. doi: 10.1111/acem.12219 24127700 PMC4038086

[10] Nguyen C; Shih J; Lin K; Aladesanmi O. Targeting National Emergency Department Overuse: A Case for Primary Care, Financial Incentives, and Community Awareness. Harvard Health Policy Rev. 2014;14(1):23–6.

[11] Shah-Canning D; Alpert J; Bauchner H. Care-seeking patterns of inner-city families using an emergency room: a three-decade comparison. Med Care. 1996;34(12):1171–9. doi: 10.1097/00005650-199612000-00002 8962583

[12] Lucas R. Ann Emerg Med. 1998 Nov;32(5):563–8. An analysis of frequent users of emergency care at an urban university hospital. http://www.ncbi.nlm.nih.gov.ezproxy.rush.edu/pubmed/9795318. doi: 10.1016/s0196-0644(98)70033-2 9795318

[13] Hunt K; Weber E; Showstack J; Colby D; Callaham M. Characteristics of frequent users of emergency departments. Ann Emerg Med. 2006;48:1–8. doi: 10.1016/j.annemergmed.2005.12.030 16781914

[14] Kraaijvanger N; VanLeeuwen H; Rijpsma D, Edwards M. “Motives for self-referral to the emergency department: a systematic review of the literature.” *BMC health services research* vol. 16,1 685. 9 Dec. 2016, doi: 10.1186/s12913-016-1935-z 27938366 PMC5148909

[15] Maugein L; Lambert M; Richer O; Runel-Belliard C; Maurice-Tison S; Pillet P. Consultations itératives aux urgences pédiatriques [Repeat visits in a pediatric emergency department]. *Arch Pediatr*. 2011;18(2):128–134. doi: 10.1016/j.arcped.2010.11.012 21215600

[16] Cunningham W. Med Care. 1999 Dec;37(12):1270–81. The impact of competing subsistence needs and barriers on access to medical care for persons with human immunodeficiency virus receiving care in the United States. doi: 10.1097/00005650-199912000-00010 10599608

[17] Douglass K; Pousson A; Gidwani S; Smith J. Postgraduate Emergency Medicine Training in India: An Educational Partnership with the Private Sector Journal of Emergency Medicine, Volume 49, Issue 5, 746–754. doi: 10.1016/j.jemermed.2015.03.010 26095219

[18] Harris P; Taylor R; Thielke R; Payne J; Gonzalez N; Conde J. Research electronic data capture (REDCap)– A metadata-driven methodology and workflow process for providing translational research informatics support, *J Biomed Inform*. 2009 Apr;42(2):377–81. doi: 10.1016/j.jbi.2008.08.010 18929686 PMC2700030

[19] Harris P; Taylor R; Minor B; Elliott V; Fernandez M; O’Neal L; et al. REDCap Consortium, The REDCap consortium: Building an international community of software partners, *J Biomed Inform*. 2019 May 9 doi: 10.1016/j.jbi.2019.103208*]*. PMC7254481 31078660

[20] Rui P; Kang K. National Hospital Ambulatory Medical Care Survey: 2017 emergency department summary tables. National Center for Health Statistics. Available from: https://www.cdc.gov/nchs/data/nhamcs/web_tables/2017_ed_web_tables-508.pdf.

[21] LaCalle E; Rabin E. Frequent users of emergency departments: the myths, the data, and the policy implications. Ann Emerg Med. 2010;56:42–48. doi: 10.1016/j.annemergmed.2010.01.032 20346540

[22] Bieler G; Paroz S; Faouzi M; Trueb L; Vaucher P; Althaus F; et al. Social and medical vulnerability factors of emergency department frequent users in a universal health insurance system. Acad Emerg Med. 2012;19:63–38. doi: 10.1111/j.1553-2712.2011.01246.x 22221292

[23] Chan C; Lin W; Yang N; Lai K; Huang H. “Pre-emergency-department care-seeking patterns are associated with the severity of presenting condition for emergency department visit and subsequent adverse events: a timeframe episode analysis.” *PloS one*. vol. 10,6 e0127793. 1 Jun. 2015, doi: 10.1371/journal.pone.0127793 26030278 PMC4452693

[24] Rao K; Shahrawat R; Bhatnagar A. (‎2016)‎. Composition and distribution of the health workforce in India: estimates based on data from the National Sample Survey. *WHO South-East Asia Journal of Public Health*, 5 (‎2)‎, 133–140. World Health Organization. Regional Office for South-East Asia. https://apps.who.int/iris/handle/10665/329660. doi: 10.4103/2224-3151.206250 28607241

[25] Tikkanen R; Osborn R; Mossialos E; Djordjevic A; Wharton G. “India: Commonwealth Fund.” *Home*, The Indrani Gupta, Institute of Economic Growth, 5 June 2020, www.commonwealthfund.org/international-health-policy-center/countries/india.

[26] Sharma M; Brandler E. Emergency medical services in India: the present and future. Prehosp Disaster Med. 2014 Jun;29(3):307–10. doi: 10.1017/S1049023X14000296 Epub 2014 Apr 10. . 24721137

[27] Issa A. Patients’ experiences and expectations from an emergency department: a survey of 4,392 patients. Middle East J. Emerg. Med. 2007; 7: pp. 57–60.

[28] Qidwai W; Ali S; Baqir M; Ayub S. Patient expectations from an emergency medical service. J. Ayub Med. Coll. Abbottabad 2005; 17: pp. 3–6. 16320785

[29] Cooke T; Watt D; Wertzler W; Quan H. Patient expectations of emergency department care: phase II–a cross-sectional survey. Can. J. Emerg. Med. 2006; 8: pp. 148–157. doi: 10.1017/s1481803500013658 17320008

[30] Harris N; Hostetler M. Parental expectations of care and charges in a tertiary care pediatric ED. Am. J. Emerg. Med. 2002; 20: pp. 601–603. doi: 10.1053/ajem.2002.35452 12442237

[31] Thompson D; Yarnold P; Williams D; Adams SL. Effects of actual waiting time, perceived waiting time, information delivery, and expressive quality on patient satisfaction in the emergency department. Ann Emerg Med. 1996 Dec;28(6):657–65. doi: 10.1016/s0196-0644(96)70090-2 . 8953956

[32] Kocher K. Effect of Testing and Treatment on Emergency Department Length of Stay Using a National Database. Acad Emerg Med. 2012 May;19(5)525–534. doi: 10.1111/j.1553-2712.2012.01353.x 22594356

