# Supplementary material for: Characteristics and expectations among emergency department patients in India
Source: PLOS Glob Public Health. 2022 Feb 11;2(2):e0000009. doi: 10.1371/journal.pgph.0000009 (PMC10021842; doi:10.1371/journal.pgph.0000009)
Supplement: S1 File — (DOCX) [file pgph.0000009.s001.docx]

You are invited to participate in a research study being conducted in coordination with George Washington University (GWU). Taking part in this research is entirely voluntary. Your decision to participate or not will not affect the medical care that you receive. If you decide not to participate, you can still receive medical from the hospital. The purpose of this study is to learn more about patient expectations and understanding of care in the emergency/casualty department. The survey you will be asked to complete will provide information that will be used to guide the future development of emergency medicine in India.

If you choose to take part in this study, you will be asked to complete two surveys, one at the beginning of you emergency/casualty department visit and one at the end of your visit. You may refuse to answer any of the questions and you may stop your participation in this study at any time. The study should take between 5 and 10 minutes to complete. Participating in this study poses no risks that are not ordinarily encountered in daily life.

You will not benefit directly from your participation in the study. The benefits to science and humankind that might result from this study are: improvements in the quality of patient care in the emergency/casualty department.

Every effort will be made to keep your information confidential, however, this cannot be guaranteed. No identifying information will be collected during this study. The study investigators will only receive the answer to your questions. If results of this research study are reported in journals or at scientific meetings, the people who participated in this study will not be named or identified.

If you have any questions please you may contact the study principal investigator at the email and phone number below.

Email: [kdavey@mfa.gwu.edu](mailto:kdavey@mfa.gwu.edu)
