# Supplementary material for: Characteristics and expectations among emergency department patients in India
Source: PLOS Glob Public Health. 2022 Feb 11;2(2):e0000009. doi: 10.1371/journal.pgph.0000009 (PMC10021842; doi:10.1371/journal.pgph.0000009)
Supplement: S3 File — (PDF) [file pgph.0000009.s003.pdf]

Strictly Confidential

MAX HEALTHCARE

Date: 18th February 2019

Ref.No. TS/MSSH/BMDRC/EM/IEC/19-02

To,
Dr. Nilesh Kumar
MEM Candidate, Department of Emergency Medicine,
Max Super Speciality Hospital, Patparganj
(A Unit of Balaji Medical & Diagnostic Research Centre)
108-A, Indraprastha Ext., Patparganj, Delhi-110092.

SUB: THESIS STUDY PROTOCOL: "PATIENT RATIONALES FOR SEEKING EMERGENCY DEPARTMENT CARE IN INDIA".

Dear Dr. Kumar,

The Institutional Ethics Committee (IEC) **[INSTITUTIONAL ETHICS COMMITTEE, MAX SUPER SPECIALITY HOSPITAL, PATPARGANJ (A UNIT OF BALAJI MEDICAL & DIAGNOSTIC RESEARCH CENTRE)]** reviewed and discussed your application for the conduct of thesis Study entitled: **"PATIENT RATIONALES FOR SEEKING EMERGENCY DEPARTMENT CARE IN INDIA",** on 11th February 2019.

The following documents were submitted by Dr. Nilesh Kumar (MEM Candidate) to the Institutional Ethics committee for review;

1. Protocol of the Study
2. Patient Information sheet & Informed consent form (English & Hindi)
3. Data Collection form
4. CV of Self, Guide & Co guide's

The following members of Institutional Ethics Committee (IEC) were present in the meeting held on Monday, 11th February 2019, between 1.30 PM – 2.30 PM at the Conference Room, Fourth Floor, Old Building, Administrative block, Max Super Speciality Hospital, Patparganj (A unit of Balaji Medical & Diagnostic Research Centre) 108- A, Indraprastha Extension, Patparganj, Delhi- 110092.

S.No.	Name	Designation	Designation As Per Schedule 'Y'	Affiliation as to the Institution (Yes/No)
1.	Prof Arun Kumar Agarwal	Medical Advisor – Education, Apollo group of Hospital's, Advisor - National Board of Examination, Senior Advisor healthcare Sector Skill council, Visiting Professor ENT- Chacha Nehru Bal Chikitsalaya, Delhi , Ex-Dean & Ex Professor of Excellence (ENT), Maulana Azad Medical College, Ex Additional Director – General, DGHS, Min. of H & FW, Govt of India.	Chairman	No
2.	Dr. Nidhi Saxena	Assistant Medical Superintendent, Max Super Speciality Hospital, Patparganj, Delhi	Member Secretary	Yes
3.	Dr. Arun Puri	Director - Department of Anesthesia & Medical Advisor, Max Super Speciality Hospital, Patparganj, Delhi,	Member (Clinician)	Yes
4.	Mr. Alok Gupta	Advocate, Supreme Court of India, New Delhi.	Legal Expert	No

Balaji Medical & Diagnostic Research Centre ETHICS COMMITTEE

Max Super Speciality Hospital, Patparganj - A Unit of Balaji Medical and Diagnostic Research Centre
(Balaji Medical and Diagnostic Research Centre Registered under the Societies Registration Act XXI of 1860)
Regd. Office: 108 A, Indraprastha Extension, Patparganj, New Delhi - 110 092
Phone: +91-11-4303 3333, Fax: +91-11-2223 5563
www.maxhealthcare.in

Letter of Hospital Support

This letter is in support of the George Washington University organized multi-centre Indian study, " **Patient Expectations of Care in the Emergency Departments in India"** with Dr. Kevin Davey as the principal investigator.

The co-investigator at our centre will be **Dr. S. Manjula Shri**. This study has been reviewed and the study protocol, survey and informed consent documents were evaluated and found that data collection can be instituted at our institution Meenakshi Mission Hospital & Research Centre.

Regards

Dr. NARENDRA NATH JENA
Director
INSTITUTE OF EMERGENCY MEDICINE
Meenakshi Mission Hospital & Research Centre
Lake Area, Madurai - 625 107, Tamilnadu

Dr. Narendra Nath Jena
Director& Head
Institute of Emergency Medicine
Meenakshi Mission Hospital & Research Centre
Madurai.

Meenakshi Mission Hospital and Research Centre
Lake Area, Madurai-625 107
4263000

F-059

Scanned by CamScanner

Believers Church
MEDICAL COLLEGE HOSPITAL

Post Box No. 31, St.Thomas Nagar, Kuttapuzha P.O., Thiruvalla - 689103

INSTITUTIONAL ETHICS COMMITTEE

Regn. No: ECR/1098/Inst/KL/2018

IEC/06/52/2019

Date: 28.02.2019

IEC STUDY Ref No.52/2019

Dr. Sumin Jacob
Department of Emergency Medicine

Dear Dr. Sumin Jacob,

Sub: Approval of Research proposal by the I.E.C

I wish to inform you that your Research Project entitled **"Patient expectations and overall understanding of services on presentation to emergency department"** has been unanimously approved by Ethical review and was approved on 28.02.2019. The approval of I.E.C is valid for a period of 2 years from the date of approval given.
You must inform the IEC of the following.

1. The occurrence of serious Adverse Events/Drug Reactions and/or Death. While conducting this Trial in the specified format.
2. Protocol amendment in the specified format.
3. (a) Discontinuation (b) Abandonment (c) Completion of this trial, stating the reasons, if the situation of 2(a) or (b) is encountered.
4. (a) It is mandatory that a 6 monthly Interim Review Report on the status of the project be submitted to the Convener in the specified format.
   (b) On completion of above Research Project- the Principal Investigator is responsible for submitting a brief summary of the results obtained, to the Convener of Institutional Ethics Committee (I.E.C) through the chairperson/Dean.

With best wishes,

Chairperson

Dr. TOMY PHILIP MD;MRCP
Reg.No: 15918
Professor of Medicine
Pushpagiri Institute of
Medical Sciences,Tiruvalla.

CC: The Assoc Director, BCMCH
The Medical Suptd, BCMCH
The HOD for file

Institutional Ethics Committee
Believers Church Medical College Hospital
St. Thomas Nagar, Kuttapuzha (P.O)
Thiruvalla - 689 103

Scanned by CamScanner
